# HealthLit4Kids study protocol; crossing boundaries for positive health literacy outcomes

**DOI:** 10.1186/s12889-018-5558-7

**Published:** 2018-06-05

**Authors:** Rose Nash, Shandell Elmer, Katy Thomas, Richard Osborne, Kate MacIntyre, Becky Shelley, Linda Murray, Siobhan Harpur, Diane Webb

**Affiliations:** 1 School of Medicine, College of Health and Medicine, University of Tasmania, Private Bag 34, Hobart, TAS 7000 Australia; 2 School of Education, College of Arts, Law and Education (CALE), University of Tasmania, Private Bag 66, Hobart, 7001 Tasmania Australia; 3 Health Systems Improvement Unit, WHO Collaboration Centre for Health Literacy, School of Health and Social Development, Faculty of Health, Deakin University, Geelong, Victoria Australia; 4 School of Medicine, College of Health & Medicine, University of Tasmania, Hobart, Tasmania Australia; 5 Peter Underwood Centre for Educational Attainment, Academic Division, University of Tasmania, Hobart, Tasmania Australia; 6 Public Health Services, Department of Health and Human Services, Hobart, Tasmania Australia; 7 Public Health Services, Department of Health and Human Services, Launceston, Tasmania Australia

**Keywords:** Health literacy, Health promotion, School, Children, Teacher, Community, Equity, Co-design

## Abstract

**Background:**

Health attitudes and behaviours formed during childhood greatly influence adult health patterns. This paper describes the research and development protocol for a school-based health literacy program. The program, entitled HealthLit4Kids, provides teachers with the resources and supports them to explore the concept of health literacy within their school community, through classroom activities and family and community engagement.

**Methods:**

HealthLit4Kids is a sequential mixed methods design involving convenience sampling and pre and post intervention measures from multiple sources. Data sources include individual teacher health literacy knowledge, skills and experience; health literacy responsiveness of the school environment (HeLLO Tas); focus groups (parents and teachers); teacher reflections; workshop data and evaluations; and children’s health literacy artefacts and descriptions. The HealthLit4Kids protocol draws explicitly on the eight Ophelia principles: outcomes focused, equity driven, co-designed, needs-diagnostic, driven by local wisdom, sustainable, responsive, systematically applied. By influencing on two levels: (1) whole school community; and (2) individual classroom, the HealthLit4Kids program ensures a holistic approach to health literacy, raised awareness of its importance and provides a deeper exploration of health literacy in the school environment. The school-wide health literacy assessment and resultant action plan generates the annual health literacy targets for each participating school.

**Discussion:**

Health promotion cannot be meaningfully achieved in isolation from health literacy. Whilst health promotion activities are common in the school environment, health literacy is not a familiar concept. HealthLit4Kids recognizes that a one-size fits all approach seldom works to address health literacy. Long-term health outcomes are reliant on embedded, locally owned and co-designed programs which respond to local health and health literacy needs.

## Background

Health literacy is the ability of an individual to find, appraise, understand and apply information to promote and maintain good health and wellbeing [1–3]. It is composed of three interwoven components; the individual, the community they belong to and the healthcare environments they access [4, 5]. This is encapsulated by Kickbusch, Wait and Maag;

*“Health literacy is the ability to make sound health decisions in the context of everyday life; at home, in the community, at the workplace, the health care system, the market place and the political arena. It is a critical empowerment strategy to increase people’s control over their health, their ability to seek out information and their ability to take responsibility.”* [6]

Some suggest health literacy is much more complex than the individual consumer and thus the terms low and high health literacy should be avoided [7, 8]. However others have demonstrated the existence of a social gradient for health literacy, reporting that financial deprivation remains the strongest predictor of low health literacy, followed by social status, education, age and gender [9].

Health literacy is influenced by both personal factors and the context in which health care encounters take place. Personal characteristics include age, social support, ability to appraise health information, educational attainment and relationships with healthcare providers. The characteristics of the health care environment include the use of plain language, signage and way-finding, and communication skills of health service providers [4]. Health literacy is dynamic as it can change over a lifetime with exposure to new or unfamiliar health settings or information.

Despite the growing literature on health literacy, there is a lack of evidence regarding effective strategies to increase health literacy, especially in children. Health attitudes and behaviours formed during childhood greatly influence adult health patterns [10], therefore it is imperative that children are supported in becoming knowledgeable and critical consumers of health information and environments. HealthLit4Kids responds to this need using a school-wide program to engage its children and their local community in conversation about health literacy and health issues. Although HealthLit4Kids is likely to have a positive impact on health outcomes, it is further justified given the positive correlation reported between health literacy and educational attainment [5, 11]. Situating health literacy education within the school context allows class teachers, who have a full appreciation of their learners’ worlds, to teach children to become increasingly involved in managing their own health. Additionally, improving the health literacy of children has the potential to trigger an intergenerational response in improving health outcomes by filtering through to families and communities an increased understanding and recall of health messages, knowledge of health determinants and use of preventive health strategies and services. When health concepts and behaviours are presented in culturally relevant, age appropriate and socially supported ways, they become normalised and children may understand their importance at an earlier-than-expected age [12].

HealthLit4Kids aims to enhance the health literacy of a whole school community through a sustainable and locally driven model. Here the whole school community includes teachers, children, support staff, school parents and friends association, parents/carers, families, local community (including health and wellbeing aligned organisations and businesses in the local area). Aligning the initiative with the Australian Curriculum recognises and supports teachers to achieve their curriculum-based objectives in addition to achieving the broader aims of HealthLit4Kids. By design, HealthLit4Kids encompasses knowledge, skills and behaviours that underpin two of the general capabilities set out in the Australian Curriculum; critical and creative thinking, and personal and social capability, and provides a mechanism to facilitate teachers and school children to explore, discuss, design and share resources capable of improving the health literacy of Australian school children and their families [13]. The recently introduced Australian Curriculum, Health and Physical Education (ACHPE) theme area provides an appropriate framework to support the sustainability of the HealthLit4Kids program. The pilot of the program (based in Tasmania, Australia) is a unique opportunity to begin to populate the ACHPE content and ensure the delivery is underpinned by health literacy design principles to maximise the benefit and outcomes of this curriculum. These curricula resources can be subsequently shared and further adapted, strengthening the synergies between education and health. HealthLit4Kids acknowledges the influence of the WHO Health Promoting Schools Framework and its elements which include: curriculum, teaching and learning, school organisation, ethos and environment and partnerships and services [14].

Structured interventions are required to improve health and equity outcomes in communities [15]. The Ophelia principles (Table 1.) can be used to underpin health literacy programs to ensure they are participatory, community-focused, equity driven and sustainable.

**Table 1:** The Ophelia (Optimising Health Literacy and Access) principles that guide the aims, development and implementation of structured interventions to improve health and equity outcomes in communities [15]

Principles	Description
1. Outcomes focused	Improved health and reduced health inequalities
2. Equity driven	All activities at all stages prioritise disadvantaged groups and those experiencing inequity in access and outcome
3. Co-design approach	In all activities at all stages, relevant stakeholders engage collaboratively to design solutions
4. Needs- diagnostic approach	Participatory assessment of local needs using local data
5. Driven by local wisdom	Intervention development and implementation is grounded in local experience and expertise
6. Sustainable	Optimal health literacy practice becomes normal practice and policy
7. Responsiveness	Recognise that health literacy needs and the appropriate responses vary across individuals, contexts, countries, cultures and time
8. Systematically applied	A multilevel approach in which resources, interventions, research and policy are organised to optimise health literacy

While much is known about the concept of health literacy and its relationship to health outcomes, there are limited studies that focus on children and the school environment [16]. HealthLit4Kids is justified given that no tools currently exist to measure the health literacy profile or competencies of children under 10 years old [17]. This program will contribute to gathering empirical evidence to identify the health literacy profile of children and determine age appropriate health literacy expectations of children. The design and implementation of HealthLit4Kids recognises the UN Convention on the Rights of the child and responds to the child’s right to participate in research about their lives [18].

It is imperative to encourage children to become engaged and knowledgeable consumers of health information and the impacts of the environments in which they learn and play. The children’s active engagement in the production of a HealthLit4Kids artefact (creative piece – poem, video, garden beds, models, painting, drawing, story, song), followed by reflection on their artefacts supports this objective. Art provides children with an age appropriate voice to express their views. HealthLit4Kids responds to a direct call to address an existent research gap whereby children’s voices and perspectives largely remain unheard. Broder et al. found that active participation by children in the conceptual development of Health Literacy was only realized in three of the 21 articles included in their recent systematic review [19]. This requires urgent attention if we are to empower a future generation of children to understand and manage their own health and wellbeing.

Improving health literacy, particularly of children, will in turn improve health outcomes by increasing understanding and recall of health messages, knowledge of the social determinants of health, and the sorts of public health actions that protect and improve health (e.g. immunisation, water as the drink of choice) and the appropriate use of preventative health services.

While it can be seen as problematic and perhaps an oversimplification of health literacy, for the purposes of this paper, the terms high and low health literacy are useful and will be used with the understanding that a more nuanced and complex context exists.

## Methods

### Methodological foundations

HealthLit4Kids reflects a pragmatist worldview, meaning the research is problem-centred, employs a real world practice orientation and inquiry approach [20]. A pragmatist position facilitates exploration of teacher awareness of health literacy and development of a school wide action plan to address the health literacy needs of a specific community. This includes greater understanding of the health literacy of individuals and the responsiveness of the school environment and community to those individuals. It seeks to better understand the health literacy profile of children.

**Fig. 1:**
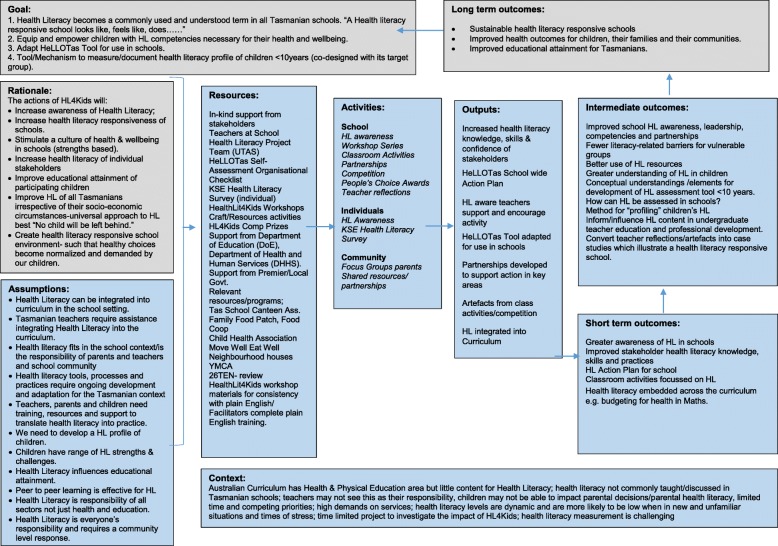
Program Logic Model

Consistent with a pragmatist approach, the research applies a sequential mixed methods strategy of enquiry [20]. It combines principles from mixed methods research, participatory action research [21], implementation science [22] and realist synthesis [23]. Figure 1 uses a program logic model [24] to visually present the program goals, rationale, resources, activities, outputs and the short, intermediate and long term outcomes.

The HealthLit4Kids program has 4 stages: needs assessment, discovery, action planning and evaluation.

**Fig. 2:**
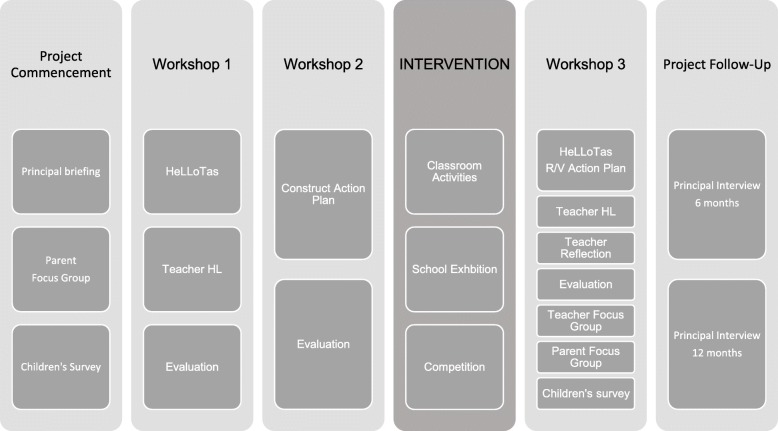
Program design including pre-post measures

The needs assessment includes the school characteristics and needs (assessed using the HeLLO Tas checklist), and the health literacy knowledge, skills and experience (KSE) of the teachers. Workshops to discuss these data lead to the co-creation of an Action Plan. Individual teachers are invited to consider their own context and explore health literacy and design individual interventions (classroom activities) to explore health and health literacy concepts through planned lessons with their class (Fig. 2).

The HeLLO Tas checklist and teacher health literacy KSE surveys are repeated at the conclusion of the program to detect if there has been any change in health literacy awareness, health literacy competencies and health literacy responsiveness. A HealthLit4Kids Competition encourages message dissemination and conversations with family and local community.

### Aims/sequence of events

As described this program is informed by a pragmatist approach and employs mixed methods design to answer nine research questions (displayed in Table 2.)

**Table 2:** Research questions mapped to methods

Research Question	Methods
1. How does a school-wide Health Literacy Project (HealthLit4Kids) affect the health literacy of the school environment?	• *Quant:* Tool 1. Self-Assessment Checklist
	• *Mixed:* Workshop evaluation (including Tool 2: Individual Health Literacy Survey)
	• *Qual:* Focus Groups (Teachers and Parents)
2. How does HealthLit4Kids affect the awareness and health literacy of the teachers involved in the project?	• *Quant:* Tool 1. Self-Assessment Checklist
	• *Mixed:* Workshop evaluation (including Tool 2: Individual Health Literacy Survey)
	• *Mixed:* Teacher 200 word reflection.
3. Of the Health and Physical Education areas outlined in the Australian curriculum which are the most commonly raised by students through their creative pieces (artefacts)?	• *Mixed:* Students’ creative pieces (artefacts)
	• *Mixed:* Competition Entry Form (artefact category/description)
	• *Mixed:* Teacher 200 word reflection.
4. How does HealthLit4Kids impact on the health literacy of the wider school community (parents, carers, community)?	• *Quant:* Tool 1. Self-Assessment Checklist
	• *Qual:* Focus Groups (Teachers and Parents)
	• *Mixed:* Teacher 200 word reflection.
5. How does feedback from teachers and students who use the Healthlit4Kids resources inform the development of a health literacy measurement tool specific for children?	• *Mixed:* Students’ creative pieces (artefacts)
	• *Mixed:* Workshop evaluation (including Tool 2: Individual Health Literacy Survey)
	• *Qual:* Focus Groups (Teachers and Parents)
	• *Mixed:* Teacher 200 word reflection.
6. What are the lessons learnt from implementation of HealthLit4kids at the trial school? How can this inform a state-wide version in the future?	• *Mixed:* Workshop evaluation (including Tool 2: Individual Health Literacy Survey)
	• *Qual:* Focus Groups (Teachers and Parents)
	• *Mixed:* Teacher 200 word reflection.
7. In what context and via what mechanisms can the HealthLIt4Kids project be optimised and sustainably embedded?	• Comparative Evaluation (using all data as per Pilot).
	• Principal Interviews – 6 months, 12 months
8. How can technology be used to optimise the reach, future participation and sustainability of HealthLit4Kids?	• A-Lab Showcase/Digital production of program and artefacts.
	• A-Lab visitor evaluation of experiential learning site.
9. How does a school-wide Health Literacy Project (HealthLit4Kids) affect children’s school engagement and attitudes and beliefs towards health behaviours?	• Student questionnaire survey based on questions in the ASHFS/CDAH survey

### Needs assessment

#### Project commencement

A briefing with the Principal provides an opportunity to determine the alignment of HealthLit4Kids with the schools existing strategic plan, to set dates for workshops, key data collection points, the school exhibition and the identification of HealthLit4Kids champions (Children, Teachers, Parents). A focus group provides an opportunity to engage the parents/carers from the commencement of the program, their contributions are fed into workshop 1. The consent forms and information sheets are sent home with children, consent is requested for use of the children’s surveys responses and the artefacts and artefact descriptions.

*Workshop 1:* This workshop defines and describes health literacy and explores its application within the school and classroom context. It gives an overview of the HealthLit4Kids aims and program design and outlines the HealthLit4Kids competition. Teachers are invited to complete an individual health literacy KSE survey to determine their baseline health literacy. The teachers are also invited as a group to define health literacy and describe a health literacy responsive school. Through a sequence of educational activities, this first workshop facilitates discussion and engagement with the concept and works towards a shared definition of health literacy. A major aspect of workshop 1 is the small group work where teachers complete the HeLLOTas tool. The tool is used to facilitate teacher led assessment of the health literacy responsiveness of the school environment. All data collected throughout the workshop informs the collaborative development of the school wide action plan. The workshop participants are also invited to complete a workshop evaluation.

### Discovery and action planning

*Workshop 2:* The second workshop supports the implementation of the action plan at a school level as well as the identification and design of individual classroom HealthLit4Kids activities (interventions). The workshop begins by revisiting the health literacy definition and description of health literate schools from workshop 1. This is a useful opportunity to carryout member checking [20], whilst also providing staff with an opportunity to add or remove items from their description. In addition to finalizing the school wide action plan teachers are provided with a template to plan their individual classroom interventions. This includes an activity description, logistical information, and asks the teachers to describe how success will be measured. The teachers are encouraged to align their individual activities to the Australian Curriculum. The authors re-introduce the idea of a school wide competition and describe how this aspect of the program brings the families, local community and surrounding business into the conversation. All participants are invited to complete a workshop evaluation.

### Intervention

Throughout Terms 3 and 4 (months August–October) teachers use their individual classroom action plans to focus on the development of HealthLit4Kids artefacts with their class. Dedicated in classroom activities support the development of artefacts in two categories- class group or individual student. The children’s description of their work accompanies each artefact. A consent form including the artefact description, age of child and the ACHPE theme area(s) is required for entry in the HealthLit4Kids competition. The teachers assist the children to identify the appropriate theme area(s). The consent forms and information sheets are sent home with children to prompt discussion about the artefact with their parents/carers. All children participate in the Classroom Activities, however entry in the competition relies on parental consent. The teachers at the pilot school chose the School Fair as the opportunity to showcase the artefacts. Parents/carers, family and friends at the fair are invited to judge the artefacts for a “People’s Choice Award”. Local business and organisations identified as Health and Wellbeing aligned are invited to donate prizes for the artefacts or consider providing activities or volunteers at the School Fair. The artefacts and their descriptions are collected and curated using the ACHPE theme areas.

### Evaluation

*Workshop 3:* The final workshop revisits the HeLLO Tas checklist to determine if there has been a change in the health literacy responsiveness of the school environment. Teachers are invited to repeat the health literacy KSE survey to allow for comparison with the survey completed during workshop 1. In addition, teachers may choose to complete a written reflection of HealthLit4Kids and their classroom intervention (to be included in their own Professional Development Portfolio) and provide this to the researchers as data. Each workshop concludes with an evaluation. In addition following workshop 3 two focus groups, one with teachers and one with parents, will be held to capture the overall impressions of the HealthLit4Kids program.

#### Project follow-up

A six month and 12 month follow up interview with the Principal and Parents & Friends will be utilised to determine the sustainability and reach of the program.

### Setting

The pilot school is situated in Southern Tasmania. Tasmania’s population has demonstrated low health literacy levels and chronic disease risk factors above the national average including smoking, obesity, physical inactivity and elevated cholesterol levels [25]. There is an imperative for improving health literacy at an individual and systems level in Tasmania, as reflected in the Premier’s ambitious target to be the healthiest state by 2020 [26]. It is within this context that HealthLit4Kids was developed with a specific focus on children and schools. The reference school has a diverse socioeconomic profile, with the majority of the population in Socio Economic Indexes for Areas (SEIFA) Decile 6 (range 5–10), indicating this is an area of medium to high socio-economic advantage relative to other areas [27]. In 2017 the school had approximately 340 enrolments, 34 teaching staff and 15 non-teaching staff. The school hosts a launch into learning program (birth to 4 years) and formal education for Kinder to Grade 6 (4-12 years).

Written consent is obtained from all participants. Written information and consent sheet are obtained from each participant to ensure consent is informed. Parental consent is provided for collection of data from children.

### Materials

Tools to measure the health literacy of individuals and environments have been developed in response to contemporary health literacy theories [4, 5]. This pilot program will use some recently developed tools to assess the health literacy environment of the school.

#### HeLLOTas Self-Assessment tool

Developed by the Tasmanian Council of Social Services (TasCOSS), the **HeLLOTas Self-Assessment tool** [28] is designed for use in health organisations within the community sector. Building on the ‘six dimensions of a health literate organisation’ developed by the New Zealand Ministry of Health [29], the HeLLoTas tool has been adapted for use in the school context as a self-rating tool to measure the health literacy responsiveness of the school environment/community. The HeLLoTas tool includes 36 questions over 6 Domains;

1. Leadership and management
2. Consumer involvement
3. Workforce
4. Meeting the needs of diverse communities
5. Access and navigation
6. Communication

The quantitative data are gathered through predesigned closed questions, with a rating scale from 1 to 5. Each of the six areas of organisational health literacy are covered. Further, participants are asked to answer two open-end questions in each of the six areas of interest. This section will provide insight into the qualitative aspect of organisational health literacy.

#### Health literacy KSE survey

This survey was designed by the Centre for Culture, Ethnicity and Health to evaluate workshops with health professionals participating in health literacy based professional development. The **Health Literacy KSE Survey** [30], includes 15 questions and a 5 point likert scale where 5 corresponds with high confidence, 1 with low confidence. Whilst many validated tools for measuring health literacy levels exist [31], this tool better reflects a more contemporary understanding of health literacy competencies; functional health literacy rather than the ability to read and understand health information alone [1, 10, 19].

**Student Survey**: adapted from the Childhood Determinants of Adult Health (CDAH) study, the seven questions were originally designed in 1989 within the Australian Schools Health and Fitness Survey which is part of a longitudinal national study and have been validated and used in previous studies that have produced numerous publications [32, 33]. The survey measures engagement, attitudes and beliefs towards health behaviour at commencement and conclusion of the project.

### Statistical analysis

#### Qualitative data

Analysis of workshop evaluations, focus group responses, teacher reflections and artefact description will employ thematic analysis [34] techniques.

#### Quantitative data

Basic statistical analysis will be employed to determine if responses in the Health Literacy KSE surveys completed by teachers at the beginning and end of the program is statistically significantly different. Given the small numbers it is anticipated the data will be non-parametric and thus non-parametric analysis will be employed. The questions and domains in the HeLLO Tas tool will also be compared pre and post to determine if there has been any change in the responses. The student survey questions will be analysed using descriptive statistics.

The few quantitative questions in the evaluation survey will also be subjected to statistical analysis using the 3 time points (each workshop) to determine if there has been a statistically significant change in agreement/response in regards to health literacy awareness and acceptance.

## Discussion

HealthLit4Kids has been designed to respect and respond to the UN Convention on the Rights of the Child (UNCRC). It embodies a salutogenic (strengths based approach to health) and through its purposive alignment with the Ophelia principles it co-creates, embeds and responds to the health literacy needs of the participating local community. Finally, by design it ensures practicality, usability and sustainability through responding to a resource gap for classroom teachers aligned to the ACHPE theme areas. Each of these design considerations are now outlined.

### UNCRC rights

Health literacy is a right of citizenship *“Just as there is a universal right of access to healthcare, the universal right of access to health literacy must be recognised.”* [6].

The Rights outlined in the UNCRC draw attention to children’s rights not only in relation to basic human needs but also in terms of research about their lives. HealthLit4Kids responds to a number of articles of the UN Convention on the Rights of the Child (UNCRC), specifically 12, 13, 17 and 24 [18]. Through purposive alignment to the UNCRC Rights HealthLit4Kids can be confident its program will assist children to;

*12. Have the right to give their opinion, and for adults to listen and take it seriously.*

*13. Have the right to find out things and share what they think with others, by talking, drawing, writing or in any other way unless it harms or offends other people.*

*17. Have the right to get information that is important to their well being, from radio, newspaper, books, computers and other sources. Meanwhile adults will ensure that the information children are getting is not harmful, and will help children to find and understand the information they need.*

*24. Have the right to the best health care possible, safe water to drink, nutritious food, a clean and safe environment, and information to help all children to stay well* [18].

### Salutogenic approach to health

HealthLit4Kids provides a salutogenic (strengths based approach to health) [35], which is now commonly utilised in Europe, particularly in the Scandinavian countries. MacDonald’s description of the benefits of this approach doubles as a useful description of the HealthLitKids program;

*“By valuing and encouraging the building upon of personal, social, community and possibly global assets and resources, students’ focus moves to how they will become educated for lifelong health and physical activity engagement and promote aspirations and action for this in their families and communities”* [36]

The decision to invite schools and teachers to be the HealthLit4Kids entry point can be justified given the risk that a program targeting parents alone may lead to disengaged parents inadvertently disadvantaging their children. This community level approach to health literacy provides all children with the opportunity to benefit from health literacy, health promotion and health programs regardless of their parents’ position on the subject. This is consistent with a universal approach to health literacy which targets all people rather than just those assessed as having low health literacy [15, 22]. Further evidence of this international movement is described by the American Heart Association which also encourages the implementation of community wide interventions that are socially and culturally appropriate to reduce disparities and inequities in cardiovascular health [37].

### Health literate schools

#### 21st century learning by doing - self awareness, critical thinking, creativity leads to empowerment

In developing the HealthLit4Kids program, multiple approaches have been considered including; Health Promoting Schools [38], Australian Research Alliance for Children and Youth (ARACY); The Common Approach [17], Head Start Communities (US) [39] and Ophelia [40].

There are concerns around the effectiveness of current approaches to school-based Health Education and increasing recognition that the traditional “one size fits all” health promotion programs imposed on schools are unlikely to be effective nor sustained beyond project funding [41, 42].

In order to realise capacity building in the school community health literacy must be meaningfully embedded in the school curriculum. As Kickbusch et al. highlight “*strategies to build health literacy must be viewed as part of life-long learning and health literacy should be integrated into the school curriculum from a young age”* [6]. Meaningfully embedding health literacy in curriculum should not come in the form of an “add on”, rather it should ensure a health literacy thread is fed throughout curriculum. For example, the development of a budget in grade 6 mathematics may encourage children to consider the costs of eating healthily and being physically active.

There is growing recognition of the importance of improving health literacy in order to improve health outcomes. However, current debate is largely confined to the health sector even though this is an issue that requires multi-sectoral collaboration and community engagement in order to achieve real and sustained progress. Few studies have engaged across sectors. Although health literacy is receiving much attention in the health sector, the education sector is well positioned to partner with health in this increasingly urgent discussion. This remains an untapped opportunity locally, nationally and internationally.

The close relationship between health literacy, educational attainment and the health behaviours of individuals highlight that a focus on health literacy is a major strategy for improving public health and reducing health inequalities [11, 43]. In addition to the potential direct benefits to the children (health outcomes and educational attainment) [5, 11] schools and curriculum [44] provide a useful point of contact with many families in the community who may not access health services nor be exposed to health initiated health literacy programs [39].

Health Literacy is listed as one of the five key propositions that underpin the ACHPE [42]. The Australian Curriculum Health and Physical Education Theme areas (which include Health Literacy) are not currently assessed formally on the A-E criteria in the Primary School setting. To date students have been awarded a Needs Attention/Acceptable/Good/Excellent for their observed ACHPE skills and behaviour. As of 2018 the formal assessment and thus moderation of student assessment items on the A-E criteria will be a requirement for all teachers. Currently there are scarce professional development, resources or example assessments to support classroom teachers with ACHPE moderation requirements. In fact, a 2015 Victorian study found that qualifications, preparation, confidence and competence of all teachers for PE implementation remained a contemporary barrier [45]. This Victorian study into the implementation of ACHPE curriculum also suggested that all teachers in the primary school require professional development as part of a “whole school” approach [45]. The HealthLit4Kids Program provides a potential solution to address this existent gap in resources and responds to the call for a whole of school approach to ACHPE.

Other innovative aspects of HealthLit4Kids include the creative development of health and health literacy inspired artefacts by schools and their children. In 2012, Paakkari and Paakari [16] recognised that health literacy involves being able to clearly communicate one’s ideas and thoughts to others. The creation of the HealthLit4Kids artefacts therefore provides the children with an opportunity to think more deeply about their health and health related decisions, put their knowledge and skills into action and use creativity and critical thinking skills to produce their final product. The artefacts which are guided by teacher’s planned learning activities and their knowledge of the children’s academic ability provide children with an age appropriate medium to express health and health literacy through their own eyes. This is justifiable given the literature supports that whilst parental views are often captured, they may not always be consistent with their child’s views [19]. Greater self-awareness is supported through the child being required to describe their artefacts which is an act of reflective learning [19]. The empowerment gained by the child through critical thinking and development of health literacy lifelong learning skills [36] are also clear advantages of this approach.

### HealthLIt4Kids

#### Collective/community level response to health Literacy and Ophelia principles

The HealthLit4Kids program is innovative in that the research seeks to explore health literacy in the classroom and seeks to capture the ripple effect the children’s classroom activities and the whole school action plan has on the children’s families and wider community. HealthLit4Kids is pioneering given that collective health literacy [19, 46] has not been carried out in the school and classroom setting in Australia before.

Given all of this, HealthLit4Kids is now justifiably explained through the lens of the 8 Ophelia principles as shown in Table 3.

**Table 3:** HealthLit4Kids alignment to Ophelia Principles

Principle	HealthLit4Kids description of alignment
1. Outcomes focused	Program Logic model (Fig. 1) describes short term, intermediate and long term outcomes. Program goals;
	1. Health Literacy becomes a commonly used and understood term in all Tasmanian schools. “A Health literacy responsive school looks like, feels like, does……”
	2. Equip and empower children with HL competencies necessary for their health and wellbeing.
	3. Adapt HeLLOTas Tool for use in schools.
	4. Tool/Mechanism to measure/document health literacy profile of children < 10 years (co-designed with its target group).
	5. Develop and populate OeR with children’s interpretations of health and health literacy.
2. Equity driven	Design is to ensure all children in the school setting are involved in discussions. A Universal approach to health literacy whereby health literacy targets all (not just those who are assessed as having low health literacy). All children despite social determinants or parents’ health literacy or health attitudes are given an opportunity to develop their own health literacy knowledge, skills and attitudes. This responds to a basic Human right and the UNCRC rights of the child.
3. Co-designed approach	At each stage (facilitated by workshops) all stakeholders/characters are involved in the development of agreed definitions, assessments, action plan and design of individual interventions.
4. Needs- diagnostic approach	Self-assessment checklist for school level health literacy responsiveness and design of tasks taking into account context, classroom, curriculum requirements, individuals and resources.
5. Driven by local wisdom	Agreed action plan focus and individual classroom activities are by teacher’s knowledge of childrens’ knowledge, skills and attitudes and the appropriate level of health literacy intervention their cohort will manage academically.
6. Sustainable	Action plan becomes part of annual cyclical review process and embedded in the school strategy and curriculum. Workshop participation and education on health literacy principles and its relevant to school context empowers teachers to implement new materials and revisit this topic with confidence in the future.
7. Responsive	The approach to co-design has portability to any context and enables diverse groups of individuals and schools to apply the same approach and potentially derive completely different goals, action plans and individual classroom activities- in response to the local context.
8. Systematically applied	Design is purposefully sequential to capture the built knowledge over time. Each stage of the Ophelia process and its corresponding workshop ensures a systematic approach to a whole of community solution to Health Literacy.

In 2017, Broder et al. reported that the extent to which families, communities and societies allow children and young people to take an active role and participate in health literacy practices remains a question for future research [19]. This is consistent with Rothman et al. who previously highlighted the importance of focussing on different opportunities for health literacy interventions including in the household and with families [47].

HealthLit4Kids is further justified given that no tools currently exist to measure the health literacy profile or competencies of children under 10 [19]. Healthlit4Kids responds to a direct call to fill the existent research gap whereby children’s voices and perspectives largely remain unheard. Being reflective of this, children’s active participation in the conceptual development process was only realized in three of the 21 articles included in the recent systematic review conducted by Broder et al. [19]. To date the focus has been on maternal or caregivers’ health literacy competencies, enabling them to secure the child’s care needs [19]. This is useful, but may not help us to challenge the intractable intergenerational health literacy barriers. DeWalt and Hink [10] describe the importance of the parent/child dyad but suggest that future research should focus on both the child and parental health literacy to determine the degree of association to health outcomes during the “transition” years (this is where the child begins to separate from the parent and take responsibility for their own health decisions). It is recognised that whilst self-care ability can be variable, children with chronic conditions usually begin to self-care from age 11 [10]. Thus identification of this transition point and empowerment prior should be a public health priority.

We come full circle then to the need to provide children with age appropriate methods for communicating their understanding of and exploring or presenting their health concerns. Abram, Klass and Dreyer report on the challenges that are inherent to establishing age-related norms or developing age related frameworks for understanding (and improving) children’s conceptualization of their own bodies, health, and health care [48]. By design, HealthLit4Kids is perfectly positioned in the school setting so that teachers can scaffold the learning and respond appropriately to the individual learner’s needs. Guidance and workshopping initially with the teachers provides them with the confidence to tackle health literacy in the classroom.

HealthLit4Kids also responds to De Walt and Hink’s [10] request for better understanding of the skills needed by children as they transition to self-management in order to inform curricula at the primary and secondary school level. As described earlier children, including primary school level or younger have not yet been at the focus of health literacy conceptual and intervention research efforts. Broder et al. provide sage advice;

*“Given that research has linked health literacy to health outcomes, and to health (care) costs for the adult population, research should follow up on past efforts in order to explore the relevance for young people as well as children”* [19].

In recognition that health literacy is not confined to the health sector, health literacy should be observed within the context that it takes place in and capture the social practices in which it is performed [19]. One such example may be the classroom supported by teachers, peers and later challenged or reinforced in the home setting. Such a comprehensive health literacy construct will be challenging to implement and operationalize. This is reflected by the necessity of a mixed methods approach to evaluating the HealthLit4Kids program. This approach is consistent with advice from Broder et al. who suggest addressing this challenge through a modular design, which is then adjusted as necessary to specific target groups, contents and contexts [19].

With all of this in mind, HealthLit4Kids seeks to;

(i) strengthen children’s and young people’s and their care takers’ personal knowledge, motivation and competences to take well-informed health decisions; and
(ii) decrease the complexity of society as a whole, and of the health care system in particular to better guide, facilitate and empower citizens, including children and young people to sustainably manage their health [19].

In the proposed rollout the involvement of health and education faculty students will ensure our future teachers and health professionals have the confidence and competence to take health literacy into the classroom and community. This boundary crossing to unite health and education using health literacy as the logical meeting point is certain to produce positive health outcomes. Thus HealthLit4Kids provides an answer to the following statement by Broder et al.

*“Future efforts must target the redesigning of systems to be inclusive and friendly towards children and young people, the adjustment of curricula and training of health professionals, teachers and other relevant stakeholders in order to better meet the challenge of the health literacy deficit, and the recognition of children and young people as active partners in their health decision making”* [19].

The HealthLit4Kids protocol outlined here contributes to the literature and our understanding of health literacy with children and their school community. It is responsive to pleas to address health literacy with children in an evidence based manner [12]. This protocol is a useful framework for other schools and their communities to discuss health literacy. Combined with the pilot findings (Completed Nov 2017) the protocol will provide clear implementation strategies to support portability of the program nationally and internationally. This protocol also has global applicability and transferability.

### Future research

Debate endures about the appropriateness of existing Health Literacy measurement tools. Currently no tools exist to measure health literacy of children below 10 years of age. This calls for the need to 1) determine if it is possible to develop a tool for children or 2) determine if it is more appropriate to describe a child’s health literacy profile.

In 2018 the project team plan to move to Stage 2 of the project and repeat the protocol design in multiple schools to determine the context, mechanisms and outcomes (CMOs) common across each and identify the factors that guarantee success and the intended outcomes of the program [40]. Comparisons may also provide greater understanding of the relationship between health literacy, SEIFA decile and educational attainment may inform more targeted solutions in the future.

Following this, the HealthLit4Kids team will need to consider appropriate methods for scalability. This will be a balancing act whereby the individualistic response to the local community and empowerment of the project participants must not be lost in the quest for efficiency usually associated with a larger scale rollout.

## Conclusion

This research protocol will provide a useful technique for other researchers that plan to explore health literacy and health literacy responsiveness in the school and classroom setting. As described in the protocol, HealthLit4Kids recognizes that crossing traditional boundaries is necessary to effect change. The protocol describes a solution to health literacy that is designed by a community in response to the specific health literacy needs of its members in their specific context. It will provide new opportunities for characters outside the health sector to contribute to awareness raising and supporting the health literacy of individuals. HealthLIt4Kids seeks to enhance the health literacy responsiveness of individuals, schools, families and communities. This multidimensional approach will translate into long term benefits. Most importantly it will provide answers to inform collective health literacy solutions. With minor modification this protocol is scalable to multiple schools and transferable globally.

## Abbreviations

ACHPE: Australian Curriculum Health and Physical Education
ARACY: Australian Research Alliance for Children and Youth
HeLLOTas: Health Literacy Learning Organisation Tasmania
HL: Health literacy
KSE: Knowledge, skills and experience
OeR: Open education resources
Ophelia: OPtimise HEalth LIteracy and Access
SEIFA: Socio Economic Indexes for Areas
TasCOSS: Tasmanian Council of Social Services
UNCRC: UN Convention on the Rights of the Child
UTAS: University of Tasmania
WHO: World Health Organisation
